# “Boys Will Be Boys” - the Medicolegal Implications of Gender Disappointment

**DOI:** 10.1192/bjo.2024.303

**Published:** 2024-08-01

**Authors:** Chrishanthy Jayarajah

**Affiliations:** Central and North West London, London, United Kingdom

## Abstract

**Aims:**

Gender disappointment can be defined as the subjective feelings of sadness when discovering the sex/gender of their child is the opposite to what the parent had hoped or expected. Wanting a boy (or “son preference” as referred to in some of the literature) has been noted for generations in many cultures, particularly in South and East Asian communities, however, is now becoming more recognised in the UK, Europe and North America.

This article aims to improve understanding of gender disappointment, as well as discuss the ethical, political and medico-legal implications of such potentially high-risk cases in clinical, forensic and social care practice.

**Methods:**

The poster reviews the key statutory literature and legal guidance in England, USA and South-East Asia specifically affecting women and girls around discussions on gender equality and reproductive rights. It also discusses high profile cases (e.g. Supreme Court decision to overturn the Roe vs Wade case) and the potential implications on reproductive health and mental well-being.

The poster also discusses the international practices influencing birth rate (such as the one child policy in China, and concerns of the dropping fertility rate in countries such as Japan), and how this, combined with deep-rooted cultural beliefs around sex and gender for preference of a son, may influence the wider socio-political discourse.

Finally the poster discusses the medico-legal and perinatal-forensic interface of gender disappointment if left unnoticed during the perinatal period, namely the risk of the possible immediate consequences of unwanted pregnancy (such a late termination, pregnancy denial and neonaticide), and the longer term risks of being an “unwanted girl” - such as violence against women and girls, forced marriage and domestic violence.

**Results:**

Gender Disappointment is a common but often missed presentation in multicultural populations. Although at present it is not identified as a distinguishable ICD-11 Diagnosis, it has the potential to impact on one's mental health during the perinatal period, and may also influence ethical and medico-legal decision making, such as in complex cases of requests for late termination of pregnancy.

**Conclusion:**

In conclusion, there is little dialogue surrounding gender disappointment which has led to misunderstanding and the potential for serious political and medico-legal repercussions and risk. My hope is that this article may act as the catalyst for a more nuanced discussion on gender issues in mental health, in collaboration with obstetric, social, forensic and criminal justice services to tackle this complex subject.

